# Exploring the associations between physical activity, self-esteem, grit, and sense of coherence among university students

**DOI:** 10.3389/fpubh.2025.1728139

**Published:** 2026-03-06

**Authors:** Ke Xu, Hongyu Jiang, Huilin Wang

**Affiliations:** 1 School of Physical Education, Hunan University of Science and Technology, Xiangtan, China; 2 School of Business, Hunan University of Science and Technology, Xiangtan, China

**Keywords:** grit, physical activity, self-esteem, sense of coherence, university students

## Abstract

**Introduction:**

University students constitute a key source of national talent, yet academic and social pressures may cause harm to their physical and psychological health. From a salutogenic perspective, sense of coherence functions as a coping resource that mitigates the effects of demanding environments. This study examines how physical activity relates to sense of coherence, considering the roles of self-esteem and grit.

**Methods:**

A cross-sectional survey used stratified cluster sampling (province × university type strata; sampled classrooms as clusters with in-class census of all eligible students). Four hundred and sixty-five valid questionnaires were collected from undergraduates in Hunan, Hubei, and Henan, China. Relationships among physical activity, self-esteem, grit, and sense of coherence were tested using structural equation modeling with maximum likelihood estimation.

**Results:**

Physical activity was positively associated with self-esteem and with grit. Self-esteem was positively associated with grit. Both self-esteem and grit were positively associated with sense of coherence. In addition, self-esteem and grit jointly mediated the relationship between physical activity and sense of coherence. Participation in physical activity appears to enhance self-esteem and grit, which in turn are linked to a stronger sense of coherence.

**Discussion:**

To promote healthier university lives, governments and universities should expand opportunities for sport and create supportive environments that encourage regular, long-term engagement in physical activity. Students are encouraged to form and maintain stable physical-activity habits.

## 1 Introduction

University students constitute a pool of future professionals whose growth can markedly shape national progress (1). However, in China, university students have faced increasing academic demands and intensified employment competition in recent years, and these stressors have been discussed as potential contributors to students’ poorer physical and mental health (2). A white paper survey on the mental health of Chinese urban residents reported that 73.6% of surveyed university students were classified as being in a sub-healthy state. In that white paper, sub-health was defined as having no clear clinical diagnosis yet experiencing persistent physical and/or psychological discomfort (3). In another survey of 7,143 university students, 0.9% (≈64) reported severe anxiety, 2.7% (≈193) reported moderate anxiety, and 21.3% (≈1,521) reported mild anxiety (4). This situation is detrimental to the country’s future development, making the promotion of university students’ physical and mental health one of the most pressing issues in the field of public health today (5). A growing body of work shows that sustained participation in physical activity is linked to a more prevention-oriented approach to health and to a stronger sense of coherence (6). However, the psychological pathways that link physical activity and sense of coherence remain to be explored in greater depth.

From a salutogenic perspective, sense of coherence is the core construct that enables individuals to comprehend demands, mobilize resources, and cope effectively, thereby dampening the impact of high pressure and demanding academic environments (7). Sense of coherence is also regarded as a resilience factor that helps university students maintain health in the face of adversity (8). Specifically, higher levels of sense of coherence are linked to stronger meaning and purpose, lower stress and anxiety, and improved quality of life, health status, and overall well-being (9). At the same time, students with a strong sense of coherence are better able to regulate their engagement in unhealthy lifestyle behaviors, thereby preventing the negative impact of such behaviors on their physical and mental health (10).

In addition, physical activity has multiple positive effects on maintaining and promoting university students’ physical and mental health (5). Specifically, physical activity can effectively reduce obesity rates among university students, thereby helping to prevent chronic diseases (11). Being physically active supports control of blood glucose, enhances cardiovascular functioning, and builds muscular capacity, which together improve general health (12, 13). Moreover, regular physical activity may buffer the impact of academic and life stressors on university students’ emotional well-being, thereby reducing the risk of negative psychological states (e.g., anxiety and depressive symptoms) and supporting subjective well-being and psychological resilience (14). Consequently, physical activity is considered a key indicator of a preventive health orientation, and changes in its intensity are associated with changes in health orientation (6). At the same time, a preventive health orientation is a determinant of sense of coherence, so individuals’ sense of coherence tends to increase as the intensity of their physical activity increases (6, 15).

Previous studies examining the relationship between physical activity and sense of coherence have mostly focused on the direct association between the two, with relatively few scholars investigating this relationship through mediating variables. In addition, research that directly addresses this link within university student populations is also limited. Much of the literature has been conducted with clinical or at-risk groups—for example people experiencing depression (16), patients with cardiovascular disease (17), and individuals who are overweight or obese (18). However, as social competition intensifies, academic pressure increases, and the employment landscape becomes more challenging, contemporary university students are also facing unprecedented psychological and physical challenges (2). Related research has shown a worsening trend in students’ mental health, with increasing rates of depression, anxiety, and self-harm (19). Thus, how to promote university students’ physical and mental health has become a major concern (5). Therefore, to fill this gap, the present study focuses on university students and aims to examine the relationship between their physical activity and sense of coherence.

Building on the salutogenic perspective advanced by Antonovsky (20)—which conceptualizes health as a continuum shaped by interacting influences where shifts in any single domain can alter overall status (21)—we further consider the roles of self-esteem and grit. Among university students, higher self-esteem can help reduce reliance on unhealthy lifestyle behaviors, lower the risk of addiction, and prevent damage to physical and mental health caused by such behaviors (22). At the same time, students with higher levels of grit are better able to cope with the anxiety arising from complex campus life and academic pressure, thereby reducing the likelihood of negative psychological states such as depression and burnout (23). Thus, within the framework of salutogenic theory, self-esteem and grit are among the key factors influencing individuals’ health status, and changes in these factors can lead to changes in overall health.

There is also a positive relationship between physical activity and both self-esteem and grit. Specifically, physical activity can effectively help university students improve their physical appearance and enhance their physical competence, thereby promoting higher levels of self-esteem (24). In addition, the process of participating in physical activity continuously exposes students to setbacks and challenges, which in turn helps cultivate their grit (25). Therefore, based on salutogenic theory, physical activity promotes self-esteem and grit while simultaneously contributing to positive developments in individuals’ overall health status. Furthermore, according to the findings of Soares-Santeugini et al. (26), sense of coherence is highly correlated with overall health. Accordingly, this study argues that interventions involving physical activity that target health-related factors among university students (such as self-esteem and grit) can further enhance their overall health status, which in turn is positively associated with sense of coherence.

At the theoretical level, by testing the mediating roles of self-esteem and grit in the relationship between physical activity and sense of coherence, this study proposes a new mediation model for understanding how physical activity influences sense of coherence. In addition, the successful application of salutogenic theory in this study extends its explanatory power from primarily physiological pathways to psychological pathways and shifts the research focus from clinical or diseased populations to healthy populations, thereby injecting new vitality into the theory’s development. This study also enriches the theoretical foundation for research on self-esteem and grit in the field of mental health and provides support for future in-depth investigations of these two variables.

At the practical level, the findings offer new ideas for university mental health education—for example, integrating elements of physical activity into curricula as an effective intervention to enhance students’ sense of coherence. They also provide theoretical support for reforms in university physical education, promoting a shift from a narrow focus on skill training toward fostering psychological well-being and personal development. Finally, these findings can offer university students a practical reference framework for self-management and personal growth.

The remainder of this paper is structured as follows: first, we review prior research and develop our hypotheses; second, we describe the research methods; third, we present the data analysis and results; and finally, we summarize the main findings and elaborate on the study’s contributions.

## 2 Literature review and hypothesis development

### 2.1 Concepts

#### 2.1.1 Physical activity

Thomas (27) characterized exercise as a social practice that is not determined by participants’ immediate attitudes or motives and that may include leisure and recreation. Building on this, and following Caspersen et al. (28), we distinguish exercise from the broader category of physical activity: exercise is a specific form of physical activity—planned, structured, and repetitive—involving bodily movement produced by skeletal muscles with associated energy expenditure, usually undertaken to improve or maintain fitness. In this tradition, exercise is also viewed as purposeful training that applies physical effort and workload for benefits related to fitness, recreation, and health (29). In line with this literature and our measurement approach, we focus on leisure-time and sport-related physical activity undertaken outside formal classes. In the present study, we use the term physical activity to refer to undergraduates’ extracurricular workouts and organized sport activities outside formal classes.

#### 2.1.2 Self-esteem

In this study, self-esteem refers to university students’ overall appraisal of their own qualities and capabilities, that is, how favorably they view themselves across important life domains. Interest in this construct has a long history. James et al. (30) offered an early quantitative idea by relating felt self-worth to the balance between one’s successes and one’s aspirations, which opened the door to measuring self-evaluation. Building on this tradition, Rosenberg (31) described self-esteem as a global evaluation of oneself that can be favorable or unfavorable. Later, scholars advanced multidimensional accounts. For example, Cast and Burke (32) conceptualized self-esteem as a person’s subjective judgment of the self with two principal facets: competence, meaning beliefs about one’s abilities and effectiveness, and worth, meaning the felt value or respect one accords to oneself. Extending this view, research has also examined domain-specific self-assessments—such as perceived intellectual ability, social skills, appearance, and athletic competence (33). Taken together, these perspectives inform our operationalization: we treat self-esteem as students’ general attitude toward themselves, integrating their characteristic self-beliefs and their perceived capability in everyday study and life.

#### 2.1.3 Grit

In this study, grit is treated as a dispositional tendency to pursue distant goals with sustained effort and stable interest. Duckworth et al. (34) introduced the construct and described it as long-run perseverance coupled with enduring passion. Following this view, many authors frame grit as multifaceted: passion denotes a lasting concern for the aims one chooses, whereas perseverance denotes the determined effort one continues to invest when difficulties arise (34, 35). Other perspectives complement this account. Gardini et al. (36) emphasize grit as a capacity to self-generate and maintain will when confronting major challenges, even without external prompts. Cassidy (37) further portrays grit as an interactive process shaped by the individual’s neuro-psychological organization and by shifting environmental conditions. Taken together, these approaches inform our operationalization. For the present research, we adopt the Duckworth et al. (34) formulation because it aligns most closely with our aims, capturing both enduring interest and persistent effort as the core features of grit.

#### 2.1.4 Sense of coherence

Drawing on Antonovsky (38), sense of coherence is a relatively enduring life orientation. It reflects the extent to which individuals perceive life demands as understandable, believe that adequate resources are available to meet those demands, and feel that investing effort is worthwhile. Olsson et al. (39) emphasize stable response tendencies when internal or external pressures arise. Hochwälder (40) further conceptualizes sense of coherence as the extent to which life is felt to be meaningful, comprehensible, and manageable. Meaningfulness refers to perceiving purpose and value in daily pursuits; comprehensibility refers to making sense of situations and information; and manageability refers to perceiving sufficient personal, social, and material resources for coping. In line with this tradition, we conceptualize sense of coherence among university students as their overall orientation toward viewing study and life demands as understandable, manageable, and worth engaging with.

### 2.2 Hypotheses

#### 2.2.1 Physical activity, self-esteem, and grit

University-based evidence indicates that regular participation in physical activity helps students cultivate health-supporting routines and attitudes, strengthens self-esteem and emotional self-regulation, and is linked to lower risks of mental-health difficulties and later chronic disease (13, 41, 42). Conversely, sustained stress and excess body weight have been associated with diminished self-esteem among undergraduates (43, 44). Physical activity offers practical countermeasures. By discouraging behaviors such as excessive drinking and unhealthy eating, it may improve body condition and appearance, enhance strength and perceived efficacy, and reduce stress (24, 45). In addition, enhancing students’ views of their own abilities and appearance is known to bolster self-esteem. Physical activity is one key route to such improvements, supporting greater body satisfaction and a more positive self-concept, which in turn relate to better mental health (46).

Neuroscientific evidence highlights the dorsomedial prefrontal cortex as central to the control processes underlying grit. Regular physical activity has been shown to increase activation in this region, suggesting a plausible link between sustained activity and higher grit (47, 48). Yu et al. (25) further argue that grit develops through gradually intensifying practice, in which people maintain long-term effort and recover from difficulties. Physical activity is a fitting arena for this development. As a self-directed lifestyle, it repeatedly exposes individuals to obstacles and setbacks, creating opportunities to practice persistence. Participation also brings physical, psychological, and social tasks that teach problem-solving and perseverance, while organized activity cultivates responsibility, teamwork, and goal setting—all of which may help build grit. Consequently, individuals who are more consistently active tend to display greater resilience than those who participate only infrequently (49).

Recent evidence shows that self-esteem and self-efficacy rise together: people who value themselves strongly hold firmer expectations about overcoming obstacles and are more prepared to devote time and effort to long-term goals (50). They also tend to fear failure less, treating setbacks not as a verdict on self-worth but as occasions to learn and grow (51, 52). Such individuals proactively adopt coping tactics, regulate emotions more effectively, and sustain motivation when pressure mounts. These tendencies overlap with the core elements of grit—long-term passion and perseverance—suggesting that individuals with higher self-esteem are more likely to exhibit greater grit.

Furthermore, the study pointed out that individuals with higher levels of self-esteem also tend to have higher self-efficacy; their beliefs about coping with setbacks are therefore more steadfast, and they are more willing to invest more time and energy to persist in achieving their goals. At the same time, argued that individuals with higher levels of self-esteem have less fear of failure because they do not treat failure as a denial of self-worth; instead, they regard failure as an opportunity for growth and learning from which they can draw lessons. Individuals with high self-esteem also actively seek coping strategies to address stress and difficulties; these strategies help them manage their emotions more effectively and maintain motivation when dealing with stress and difficulties, thereby facing setbacks optimistically and sustaining long-term commitment to their goals. In addition, individuals with strong self-esteem possess stronger social adaptability and, when solving problems, show greater resilience than those with low self-esteem; their fear of difficulties and setbacks is also relatively weaker. In summary, building on prior perspectives, this study proposes the following hypotheses:

*Hypothesis 1 (H1)*: Physical activity is positively correlated with self-esteem.

*Hypothesis 2 (H2)*: Physical activity is positively correlated with grit.

*Hypothesis 3 (H3)*: Self-esteem is positively correlated with grit.

#### 2.2.2 Self-esteem, grit, and sense of coherence

Moksnes and Lazarewicz (53) report that a strong sense of coherence is linked with favorable health appraisals, including better mental health and quality of life, and they note that self-esteem operates as a protective resource that attenuates harmful influences and supports both physical and mental well-being. Complementing this view, Świtaj et al. (54) argue that developing a sense of coherence depends on resistance resources—personal, social, and material assets that help people grasp life, manage demands, and find meaning. Among these resources, self-esteem is prominent; accordingly, higher self-esteem forecasts a higher sense of coherence. Individuals who value themselves more tend to approach problems with greater assurance and are more inclined to view life as manageable (55). This positive self-understanding may also support clearer interpretation of the environment and stronger affirmation of self-worth, thereby promoting engagement with life and higher life satisfaction (55, 56).

When people display greater grit, they tend to read life’s meaning more deeply, feel more agency over their direction, and make sounder judgments about difficulties (57). In practice, grit cultivates feelings of accomplishment and satisfaction during goal pursuit, which reinforces one’s sense of life meaning (58, 59). Gritty individuals also actively mobilize resources to confront challenges, thereby improving how they manage life (60). Through repeated effort and learning, they may also develop a clearer understanding of setbacks and the surrounding context (61). Related work shows that sense of coherence aligns with resilience, and that grit helps sustain long-term passion and perseverance in adverse conditions, buffering harmful influences; consequently, higher grit is typically associated with stronger resilience (62). Building on these insights, the present study advances the following hypotheses:

*Hypothesis 4 (H4)*: Self-esteem is positively correlated with sense of coherence.

*Hypothesis 5 (H5)*: Grit is positively correlated with sense of coherence.

#### 2.2.3 The mediating effect

Regular participation in physical activity improves cardiorespiratory fitness, motor functioning, and overall physical condition. These improvements are often accompanied by a stronger sense of self-worth and, consequently, higher self-esteem (22). Physical activity also discourages behaviors such as excessive drinking and unhealthy eating, which can enhance physique and body satisfaction. As satisfaction with one’s body increases, self-esteem typically rises as well (24, 46, 63). In addition, engaging in physical activity entails sustained pursuit of long-term aims (64). Because participants must repeatedly adjust strategies, refine techniques, and persist despite setbacks, physical activity may foster grit over time (25, 65).

From a salutogenic standpoint, health is an integrated system shaped by multiple influences; shifts in any of these influences—such as self-esteem and grit—can alter overall health (21, 53, 66). Individuals with higher self-esteem tend to view difficulties as predictable and interpretable, which supports a coherent understanding of life and reduces stress, anxiety, and other harmful effects on physical and psychological well-being (67, 68). Likewise, people with higher levels of grit are more likely to maintain healthy dietary routines, moderate total caloric intake, and thus reduce risks of excessive obesity and chronic illness, promoting better physical and mental health (69, 70). Consistent with Soares-Santeugini et al. (26), overall health status is closely related to sense of coherence. Accordingly, we posit a mediating hypothesis: improvements in self-esteem and grit constitute key psychological pathways through which physical activity relates to sense of coherence. Taken together, these arguments imply that self-esteem and grit function as health-related psychological resources that channel the beneficial effects of physical activity into a stronger sense of coherence.

Accordingly, building on prior perspectives, this study proposes the following hypothesis:

*Hypothesis 6 (H6)*: Self-esteem and grit mediate the association between physical activity and sense of coherence.

**Figure 1:**
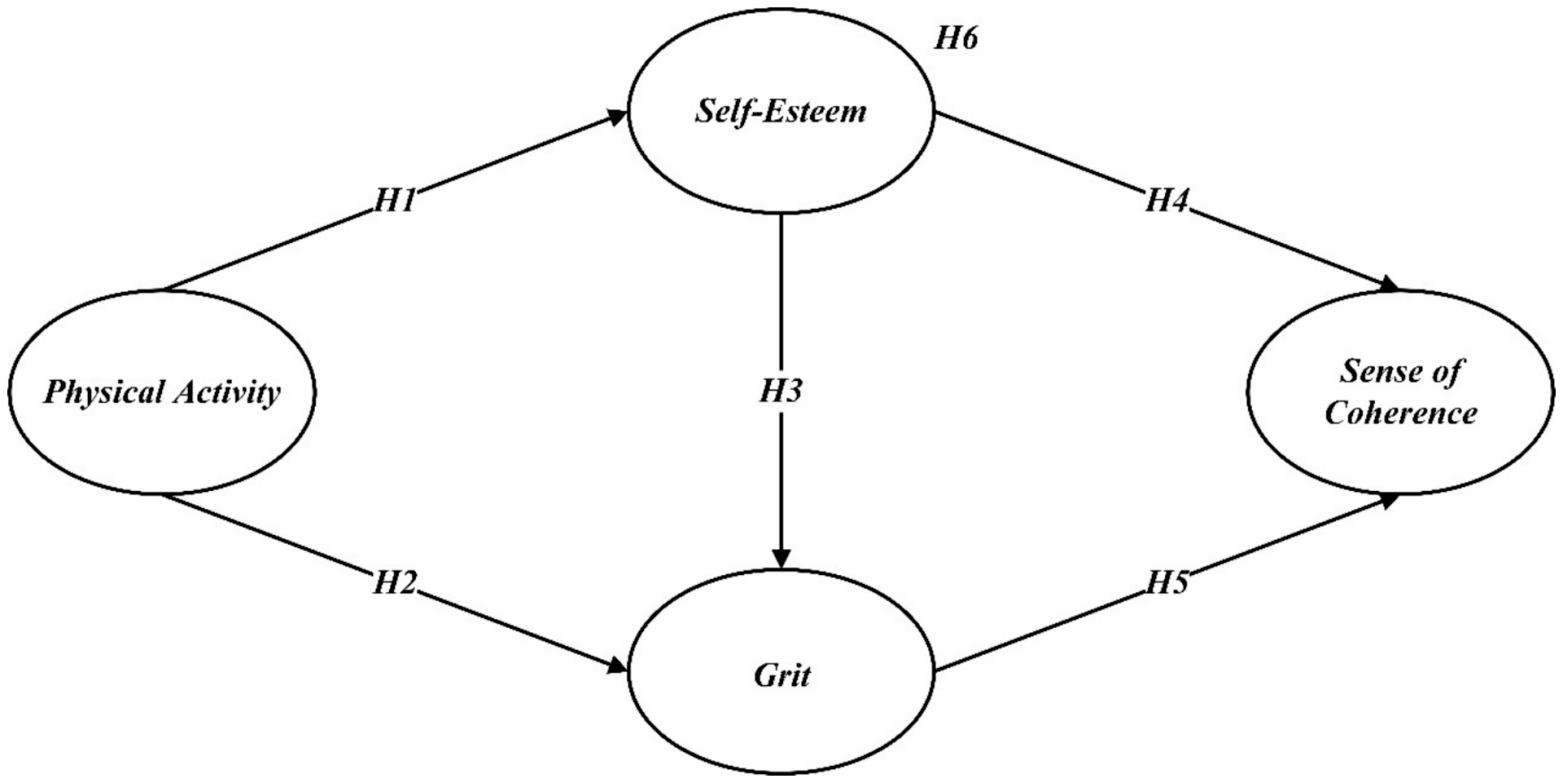
Conceptual model.

Figure 1 summarizes the proposed hypotheses.

## 3 Methods

### 3.1 Participants and procedures

We conducted a cross-sectional survey using a stratified cluster sampling design among currently enrolled undergraduates at universities in Hunan, Hubei, and Henan, China. Universities were stratified by province and type to ensure representativeness across provinces and university types; within selected universities, large compulsory general education courses and university-wide public elective (“public”) courses were sampled as classroom clusters, and all students present who were eligible were invited (census within cluster). Eligibility criteria were being currently enrolled as an undergraduate at the university and being able to complete the questionnaire independently. Paper questionnaires were administered on site by trained research assistants. Participants provided written informed consent prior to completing the survey. Questionnaires were completed in the classroom in the presence of trained research assistants (not course instructors) to minimize discussion and ensure independent responses; participation was entirely voluntary and responses were recorded anonymously. As a minimal token of appreciation, participants who completed the survey received a small gift (e.g., a low-value voucher or pool pass); the incentive was of negligible value and was not expected to unduly influence participation.

We distributed 550 paper questionnaires; after excluding questionnaires with substantial missing data or uniform response patterns, 465 usable questionnaires remained, yielding a valid response rate of 84.5%. The final sample was 52.0% male (*n* = 242) and 48.0% female (*n* = 223); approximately half were first- or second-year students, and most majored in science/engineering or humanities, with the vast majority residing on campus. Descriptive characteristics of these 465 participants are summarized in Table 1.

**Table 1:** Sample characteristics.

Profiles	Options	Frequency	Survey (%)
Gender	Male	242	52.0
	Female	223	48.0
Grade	Undergraduate (year 1–2)	217	46.7
	Undergraduate (year 3–4)	171	36.8
	Postgraduate (master’s or above)	77	16.6
Major	Humanities	165	35.5
	Science and engineering	217	46.7
	Arts and sports	54	11.6
	Others	29	6.2
Do you live on campus?	Yes	427	91.8
	No	38	8.2

### 3.2 Instruments

The questionnaire comprised five sections. Section one gathered demographics: gender, year of study, academic field, and on-campus versus off-campus residence.

Section two assessed physical activity using three items from the Chinese revision of the exercise scale by Liang (71), which has been widely applied in Chinese university samples [e.g., Xu et al. (42)]. The scale includes one item each for typical intensity (“What is the intensity of physical activity that you usually participate in during the past month?”), duration (“How long do you spend in each physical activity session?”), and frequency (“How often do you do physical activity every month/week?”). Each item is rated on a five-point scale (1–5), with higher scores indicating higher levels of that dimension. This brief, three-item Chinese-language scale was chosen because it aligns well with China’s cultural and exercise context, has been widely applied in prior research with Chinese samples, and has demonstrated acceptable reliability and validity in those studies (42, 71).

Section three measured self-esteem with five positively worded items from the self-esteem scale adapted by Wood et al. (72), (sample item: “I feel I do have much to be proud of”). Responses used a five-point Likert scale (1 = strongly disagree … 5 = strongly agree); higher scores reflect higher self-esteem. All items were summed and averaged to obtain a composite self-esteem score.

Section four measured grit with eight items drawn from the two facets of the Grit Scale developed by Duckworth and Quinn (73) (sample item: “Setbacks do not discourage me”). Items were rated on the same five-point Likert scale; higher scores indicate higher grit. In this study, items were combined into a single overall grit score by averaging across all eight items.

Section five used the sense of coherence scale revised by Jasiński et al. (74), covering meaningfulness, comprehensibility, and manageability (sample item: “My life has very clear goals and meaning”). Participants responded on a five-point scale from 1 (never) to 5 (always); higher scores denote a stronger sense of coherence. An overall sense of coherence score was computed by averaging all items, with higher values indicating a stronger sense of coherence.

Before the main survey, we ran a pilot and obtained 95 valid questionnaires. Across these pilot data, Cronbach’s alpha values for all multi-item scales exceeded 0.80, providing preliminary evidence of strong internal consistency and supporting the suitability of the revised instruments for use in the main study.

### 3.3 Data analysis

We analyzed the hypothesized relations using structural equation modeling together with partial least squares structural equation modeling to cross-check results. This approach is well suited for testing links among latent constructs and for evaluating both measurement and structural components (75). Adopting a two-step strategy (76), we first assessed the measurement model to establish reliability and validity; the lowest Cronbach’s alpha observed in this stage was 0.847, indicating excellent consistency. We then estimated the structural model using maximum likelihood estimation to examine paths among physical activity, self-esteem, grit, and sense of coherence. To evaluate indirect effects, we generated 5,000 bootstrap samples. Model quality was judged by global fit indices and standardized path coefficients for the proposed model. The design, analysis, and reporting of this cross-sectional study followed widely accepted reporting guidelines for cross-sectional studies.

## 4 Results

### 4.1 Measurement model

We evaluated the latent constructs with confirmatory factor analysis in SmartPLS. All scales showed Cronbach’s alpha values above 0.80 (Table 2), indicating strong internal consistency and meeting the criteria proposed by Fornell and Larcker (77). Convergent validity was supported on several fronts: the AVE for every construct was greater than 0.60 (Table 2), the CR of each construct exceeded 0.80, and the standardized factor loadings from the principal-component solution ranged from 0.846 to 0.972 (Table 2). Together, these results demonstrate a well-specified measurement model with robust reliability and convergent construct validity.

**Table 2:** Loadings, α, CR, AVE.

Items	Factor loadings	Cronbach’s *α*	CR	AVE
Physical Activity (PA)		0.847	0.908	0.766
PA1	0.878			
PA2	0.901			
PA3	0.846			
Self-Esteem (SE)		0.980	0.984	0.927
SE1	0.950			
SE2	0.958			
SE3	0.970			
SE4	0.967			
SE5	0.968			
Grit (GR)		0.981	0.983	0.881
Consistency of Interest		0.966	0.975	0.907
CI1	0.936			
CI2	0.960			
CI3	0.962			
CI4	0.950			
Perseverance of Effort		0.968	0.977	0.913
PE1	0.949			
PE2	0.950			
PE3	0.959			
PE4	0.964			
Sense of Coherence (SC)		0.980	0.982	0.913
Meaningfulness				
ME1	0.966			
ME2	0.969			
ME3	0.972			
Manageability		0.929	0.955	0.875
MA1	0.918			
MA2	0.945			
MA3	0.943			
Comprehensibility		0.958	0.973	0.922
CO1	0.957			
CO2	0.969			
CO3	0.955			

Cα, Cronbach’s alpha; AVE, average variance extracted; CR, composite reliability.

Following Fornell and Larcker (77), discriminant validity is supported when the square root of each construct’s AVE (values on the diagonal) exceeds its correlations with other constructs. Factor loadings, Cronbach’s alpha, AVE, and CR are reported in Table 3, and the discriminant validity matrix is presented in Table 3. Taken together, these statistics show that the measurement model is sound and suitable for estimating the structural relations in the final analysis.

**Table 3:** Discriminant validity matrix.

Variable	Grit	Physical activity	Sense of coherence	Self-esteem
Grit	**0.939**			
Physical activity	0.586	**0.875**		
Sense of coherence	0.786	0.496	**0.928**	
Self-esteem	0.733	0.427	0.783	**0.963**

Bold values on the diagonal represent the square roots of the average variance extracted (AVE) for each construct.

### 4.2 Structural model

Model adequacy was evaluated with the standardized root mean square residual (SRMR), which indexes the discrepancy between observed and model-implied covariances; values at or below 0.10 are typically viewed as acceptable (78). The estimated SRMR was 0.030, indicating a satisfactory overall fit.

**Figure 2:**
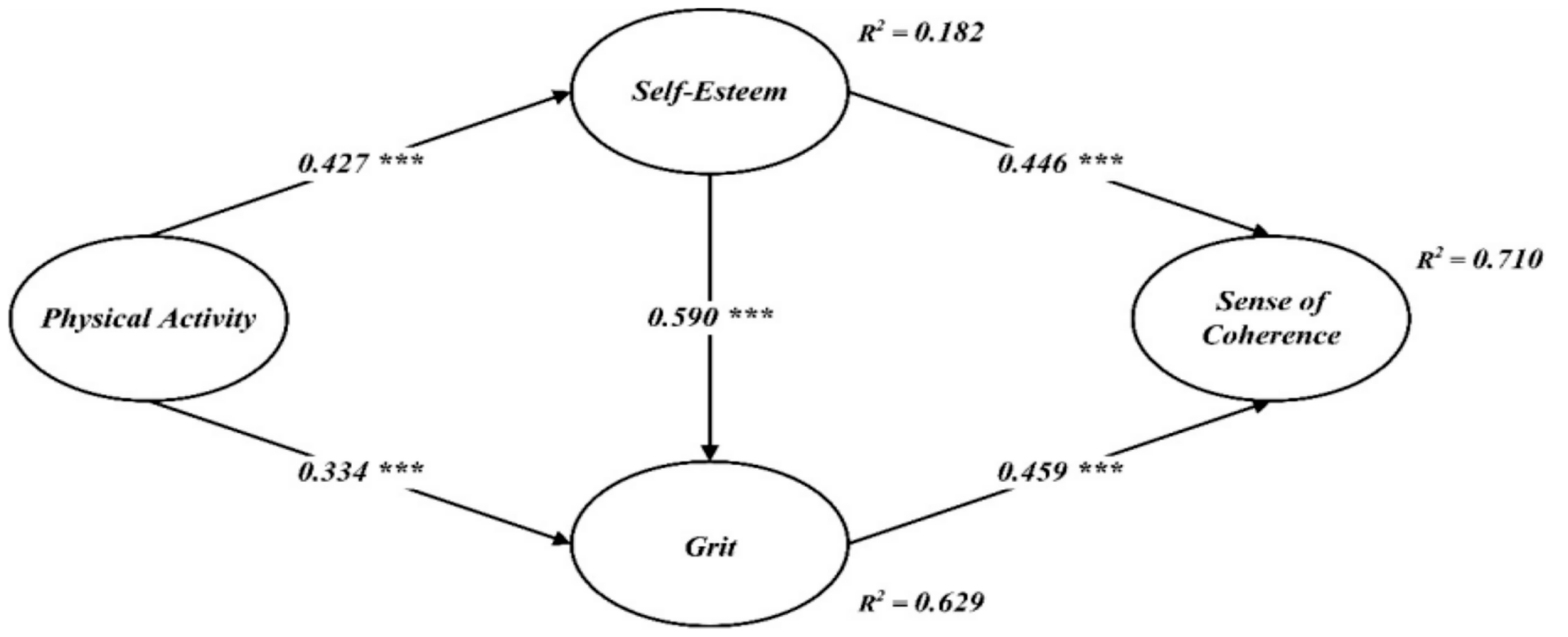
Structural path model. ***indicates statistical significance at the 0.001 level (**p < 0.001).

Turning to the structural relations, all hypothesized paths were positive and statistically significant (see Table 4 and Figure 2). Physical activity predicted higher self-esteem (*β* = 0.427, *t* = 11.157, *p* < 0.001) and higher grit (*β* = 0.334, *t* = 7.917, *p* < 0.001), consistent with H1 and H2. Self-esteem, in turn, was associated with greater grit (*β* = 0.590, *t* = 13.002, *p* < 0.001), supporting H3. Moreover, self-esteem related positively to sense of coherence (*β* = 0.446, *t* = 5.972, *p* < 0.001) and grit also showed a positive link with sense of coherence (*β* = 0.459, *t* = 6.037, *p* < 0.001), thereby supporting H4 and H5. Collectively, these coefficients align with the proposed model and indicate that the pattern of effects among physical activity, self-esteem, grit, and sense of coherence is ell captured by the specified structure.

**Table 4:** Path coefficients.

No.	Path	*β*	Mean	*T* statistics	*p*-values	LLIC	ULIC
H1	PA → SE	0.427	0.427	11.157	0.000	0.350	0.499
H2	PA → GR	0.334	0.335	7.917	0.000	0.250	0.418
H3	SE → GR	0.590	0.590	11.214	0.000	0.500	0.679
H4	SE → SC	0.446	0.442	5.972	0.000	0.291	0.583
H5	GR → SC	0.459	0.463	6.037	0.000	0.321	0.618

GR, grit; PA, physical activity; SC, sense of coherence; SE, self-esteem.

### 4.3 Mediation analysis

We gauged the model’s explanatory strength by inspecting the *R*^2^ values of the endogenous variables, which reflect the proportion of variance accounted for by the structural paths (79). Following Falk and Miller (80), *R*^2^ ≥ 0.10 was taken as an acceptable benchmark. As reported in Table 5, all endogenous constructs met this criterion, indicating adequate in-sample predictive capacity. We also assessed predictive relevance using the Stone–Geisser *Q*^2^ statistic obtained via blindfolding; *Q*^2^ > 0 signals that the model has out-of-sample predictive utility. The values in Table 5 confirm meaningful predictive performance for the specified constructs.

**Table 5:** *R*^2^ and *Q*^2^ indices.

Variable	*R* ^2^	Adjusted *R*^2^	*Q* ^2^
GR	0.629	0.627	0.550
SC	0.710	0.709	0.607
SE	0.182	0.181	0.168

GR, grit; SC, sense of coherence; SE, self-esteem.

We theorized that physical activity influences sense of coherence via two mediators—grit and self-esteem. Mediation was evaluated with bias-corrected bootstrapping (81). As shown in Table 6, both grit and self-esteem carried the effect of physical activity to sense of coherence; the standard indirect effect = 0.460, *p* < 0.001, thereby supporting H6.

**Table 6:** Mediation results.

No	Path	Original sample (O)	Sample mean (M)	Standard deviation (STDEV)	T statistics (|O/STDEV|)	*P*-values
H6	Total indirect effect of PA → SC	0.460	0.461	0.033	13.788	0.000
	Total Effect of PA → SC	0.460	0.461	0.033	13.788	0.000

PA, physical activity; SC, sense of coherence.

## 5 Discussion

### 5.1 Theoretical contributions

Salutogenic thinking has been taken up across health promotion and clinical settings. For instance, Shorey and Ng (82) and Seah et al. (83) illustrate how gathering patients’ life histories and viewing their condition through a salutogenic lens can guide nursing strategies that prevent harm. Building on this perspective, our study examines four constructs that matter for university students’ overall health: physical activity, self-esteem, grit, and sense of coherence.

As employment and academic pressures have surged in contemporary society, the physical and mental health of university students has become increasingly concerning (2). For example, Li et al. (84) found that 3–4 out of every 10 university students met criteria for depression. Yuting and Rashid (19) reported a rising trend in mental health problems among today’s students, including anxiety, suicidal ideation, and self-harm. At the same time, shifts in students’ lifestyles have contributed to higher obesity rates and lower levels of grit in this population (11, 85). Obesity, in turn, can impair students’ body shape and physical functioning, thereby lowering their self-esteem (46). Thus, identifying ways to enhance and maintain university students’ physical and mental health has become a major and urgent challenge. Our analyses show that physical activity relates positively to both self-esteem and grit, and that these two psychological resources carry the association from physical activity to sense of coherence. This pattern accords with the salutogenic premise that health constitutes a dynamic whole shaped by multiple determinants and that a shift in one domain can alter the wider system (21).

More specifically, within the framework of salutogenic theory, physical activity promotes positive development in key health-related factors—such as self-esteem and grit—which in turn drives positive change in overall health among university students (21, 63, 65). Prior research has also shown that self-esteem and grit can function as predictors that exert a positive influence on sense of coherence (55, 59). For instance, Rasool et al. (56) and Pomfret et al. (61) found that individuals with higher levels of self-esteem and grit tend to experience a stronger sense of meaning, better cognitive manageability, and greater comprehensibility in their lives. Building on salutogenic theory and drawing on the perspective of Soares-Santeugini et al. (26), the present study further demonstrates that, among university students, self-esteem and grit mediate the relationship between physical activity and sense of coherence. In other words, by enhancing students’ self-esteem and grit, physical activity contributes to improvements in their overall health and sense of coherence (54, 62, 68, 69).

Secondly, our finding of a positive association between physical activity and self-esteem contrasts with Bean et al. (86), who argued that very high volumes of physical activity can heighten psychological stress and, as a result, undermine mental health and self-esteem. This discrepancy likely stems from differences in research context. Bean et al. (86) focused primarily on participants engaged in competitive sports, whose main goals in being physically active were to achieve better competition results and to enhance their athletic performance. To reach these goals, they often engaged in very high-intensity exercise, which can lead to excessive physical load, fatigue, or injury. These adverse physical consequences may spill over into daily life, resulting in performance in everyday activities, training, or academics that falls short of expectations, thereby elevating psychological stress and lowering self-esteem (86).

In the present study, physical activity was measured using three items from Liang (71) Chinese exercise scale, capturing typical intensity, duration, and frequency of extracurricular physical activity during the past month. Our instructions defined physical activity as leisure-time and sport-related activity undertaken outside formal classes. Thus, our physical activity variable reflects students’ general out-of-class participation rather than intensive, performance-focused training. While our measure does not directly distinguish leisure from performance-driven engagement, the context of typical undergraduates suggests that most reported activity is leisure-oriented and self-paced. This may help explain why we observed a positive association with self-esteem.

We also detected a positive relation between physical activity and grit. Importantly, grit in this context does not require competitive mastery. Even leisure activities (e.g., walking, jogging, recreational fitness) can foster grit when they are pursued as sustained routines with specific, progressive goals. Maintaining regular sessions, tracking progress, and persisting despite time constraints, boredom, or setbacks can provide repeated practice in perseverance. This result aligns with the conclusions of Yu et al. (25) and Nothnagle and Knoester (49), who argue that participation in physical activity can effectively train individuals’ capacity for sustained effort.

In addition, the present study found a positive association between self-esteem and sense of coherence. This pattern is consistent with prior work suggesting that self-esteem functions as a resistance resource that supports sense of coherence (54). Specifically, individuals with higher self-esteem typically display greater confidence, organize their lives more effectively, and maintain more favorable self-views; consequently, they are less easily unsettled by environmental pressures (55). Self-esteem also helps individuals recognize their own value more clearly, making it easier for them to clarify the meaning and goals of their lives, which in turn boosts their engagement with and satisfaction in life (56).

At the same time, we also found a positive association between grit and sense of coherence. This result is in line with the argument of Vainio and Daukantaitė (62) that levels of sense of coherence are closely related to how resilient individuals are when facing adversity. More specifically, grit also acts as a motivational driver: it facilitates feelings of accomplishment and satisfaction amid difficulty, reduces distress in the face of challenges and setbacks, and supports sustained passion and perseverance over extended periods (58, 60, 62). Grit can also prompt individuals to gain a deeper understanding of the nature of difficulties and setbacks, thereby enhancing the comprehensibility component of sense of coherence (61).

### 5.2 Practical implications

In campus life, university students are exposed to multiple academic, social, and employment-related stressors. Our findings indicate that higher levels of physical activity are associated with a stronger sense of coherence, and that this link is partly carried by self-esteem and grit. These results suggest that practical efforts to protect students’ physical and mental health should not only increase opportunities for physical activity, but should also deliberately use physical activity as a vehicle to foster self-esteem and perseverance. On this basis, we propose concrete recommendations at the policy, university, and individual levels.

At the policy level, public authorities could prioritize investments that explicitly link physical activity with student mental health promotion. Dedicated funds may be allocated to build and upgrade inclusive, everyday-use sports facilities (e.g., walking tracks, multi-purpose courts, fitness corners) that support moderate, sustainable participation rather than only competitive training. Governments can subsidize campus-based programs that combine recreational physical activity with structured opportunities to experience mastery (e.g., progressive skill courses, step-by-step training plans), thereby strengthening students’ self-esteem and grit. In addition, policy makers may encourage universities to pilot and evaluate integrated “physical activity + psychological resource building” projects, and disseminate effective models through guidelines and best-practice documents.

At the university level, institutions can translate these findings into concrete campus practices. Universities may increase support for student sports clubs and societies, while guiding them to emphasize encouragement, mastery experiences, and persistence rather than only performance outcomes, so as to build self-esteem and grit through participation. Physical activity can be embedded into timetables and general education requirements (e.g., credit-bearing courses focusing on regular moderate exercise with goal-setting and reflection tasks). Opening hours and booking systems for sports venues can be adjusted to make after-class activity more convenient, and PE instructors and club leaders can be trained to provide autonomy-supportive feedback that recognizes effort and progress, thereby reinforcing students’ sense of competence and long-term commitment.

At the individual level, students can be encouraged to view physical activity not only as a way to “keep fit” but also as a means to strengthen their self-esteem, grit, and sense of coherence. Practically, this includes setting realistic and specific activity goals (e.g., number of sessions per week), tracking their own progress, and acknowledging small improvements in skills or endurance to build confidence. Students are advised to choose activities they enjoy and can sustain over time, to schedule regular sessions after class, and to participate in groups or clubs that provide social support and positive feedback. Where possible, counseling or mental health services can collaborate with sports units to recommend suitable forms of physical activity as part of self-management plans for students facing stress or reduced psychological resources.

### 5.3 Limitations

The study drew participants through snowball and convenience approaches in universities located in three provinces of central China. Although these non-probability strategies simplify fieldwork, they can introduce selection bias and restrict population coverage, which in turn narrows external validity. Moreover, the geographic scope of data collection was restricted to three central provinces, which may further limit the generalizability and representativeness of the findings. Representativeness therefore remains limited due to the combined use of non-probability sampling methods and restricted regional coverage. Subsequent research should employ probability-based sampling (for example, stratified or cluster random sampling) and widen the geographic catchment to improve coverage and strengthen the generalizability of the findings.

A second limitation is the cross-sectional design. Observations at a single time point do not establish temporal ordering and therefore cannot support strong causal claims. Future studies could use longitudinal panels or intervention-based designs to follow individuals over time, repeatedly measuring physical activity, self-esteem, grit, and sense of coherence. Such designs would allow researchers to trace within-person change, test mechanisms more rigorously, and capture trends and transitions in these relationships.

A third limitation concerns the use of self-report questionnaires among university students. All key variables were measured via self-report, which may be subject to recall bias and social desirability bias and may inflate associations due to common method variance. Future research could combine self-report scales with more objective indicators (for example, wearable-device data for physical activity or external records where appropriate) to obtain more reliable measurements and enhance the robustness of the findings.

## 6 Conclusion

The results indicate a coherent pattern: among university students, physical activity, self-esteem, grit, and sense of coherence are all positively linked, and participation in physical activity appears to strengthen sense of coherence indirectly through higher self-esteem and greater grit. On this basis, we recommend that students cultivate regular physical activity to reinforce their sense of coherence and to buffer the adverse pressures of campus life, thereby supporting healthier university experiences. We further urge governments and universities to expand investment in sporting provision and to build a supportive exercise culture—measures that can lower barriers to participation, encourage sustained engagement, and facilitate students’ healthy progression through university.

## Data Availability

The raw data supporting the conclusions of this article will be made available by the authors, without undue reservation.
